# Second lung malignancy and Richter syndrome in chronic lymphocytic leukemia: case report and literature review

**DOI:** 10.1186/s40248-017-0107-2

**Published:** 2017-09-29

**Authors:** Ghassen Soussi, Selsabil Daboussi, Samira Mhamdi, Zied Moatemri, Hela Ghedira, Chiraz Aichaouia, Mohsen Khadhraoui, Faouzi El Mezni, Rezaik Cheikh

**Affiliations:** 1 Department of Respiratory Medicine, Military Hospital of Instruction of Tunis, Tunis, Tunisia; 2 Department of Hematology, Military Hospital of Instruction of Tunis, Tunis, Tunisia; 3 Department of Pathology, Abderrahmen Mami Hospital, Ariana, Tunisia

**Keywords:** Chronic lymphocytic leukemia, Richter syndrome, B cell lymphoma, Second lung malignancy, Lung cancer, Lymphadenopathy

## Abstract

**Background:**

Chronic lymphocytic leukemia (CLL) is the most frequent lymphoproliferative disease. Transformation into Richter disease and occurrence of second malignancies involving the lungs are rare complications. The hallmarks of any thoracic involvement are still unknown.

**Case presentation:**

We report a case of a 56-year-old male patient, with history of tobacco smoking, who presented with recurrent hemoptysis, fatigue and weight loss. Physical examination was normal except a slightly enlarged supraclavicular lymph node. Chest x-ray revealed a mediastinal widening due to enlarged paratracheal nodes and a left parahilar infiltrate. Blood tests showed a hyperlymphocytosis and a biological inflammatory syndrome. CT scan showed bilateral mediastinal and axillary lymphadenopathy, as well as left supraclavicular lymphadenopathy, with a left upper lobe alveolar attenuation and a solitary contralateral pulmonary nodule. Examination of Virchow’s node and bone marrow biopsies confirmed metastasis of a pulmonary adenocarcinoma, as well as chronic lymphocytic leukemia with Richter’s transformation. The clinical course was unfavorable since the first days of therapy as the patient passed away in a matter of a few days.

**Conclusions:**

Steady surveillance of CLL patients and systematic screening for second solid tumors, particularly lung cancer, and Richter’s transformation seem to be relevant more than ever. Early diagnosis might help us understand the pathways leading to these complications and adapt therapy.

## Background

Determinism of lymphoproliferative disorders involves a complex interplay of many underlying processes, taking place at different scales both in space and time. When the normal mechanisms of control of proliferation of lymphocytes break down, shake-ups in the immune system functioning might pave the way for solid tumors to surge in an environment of immune tolerance, conducive to malignancy.

We hereby report a rare case of chronic lymphocytic leukemia (CLL) with Richter’s transformation, bringing about a second lung malignancy.

## Case presentation

A 56-year-old caucasian male patient, with history of active cigarette smoking (35 pack/years), hypertension and coronary heart disease (for which he underwent a double coronary artery stenting), presented with recurrent hemoptysis of mild abundance which first occurred no less than 4 months before. He also reported fatigue and weight loss amounting to 6 kg in 4 months. There was no associated history of fever, night sweats or extra-thoracic symptoms and no household contact of tuberculosis.

Upon admission, the physical examination showed a patient in a good condition (performance status 1) with no fever and no chest abnormalities except for few rhonchi on lung auscultation and a slightly enlarged supraclavicular lymph node. There was no clubbing, no extra-thoracic signs and no other superficial lymph nodes meeting size criteria for lymphadenopathy.

**Fig. 1:**
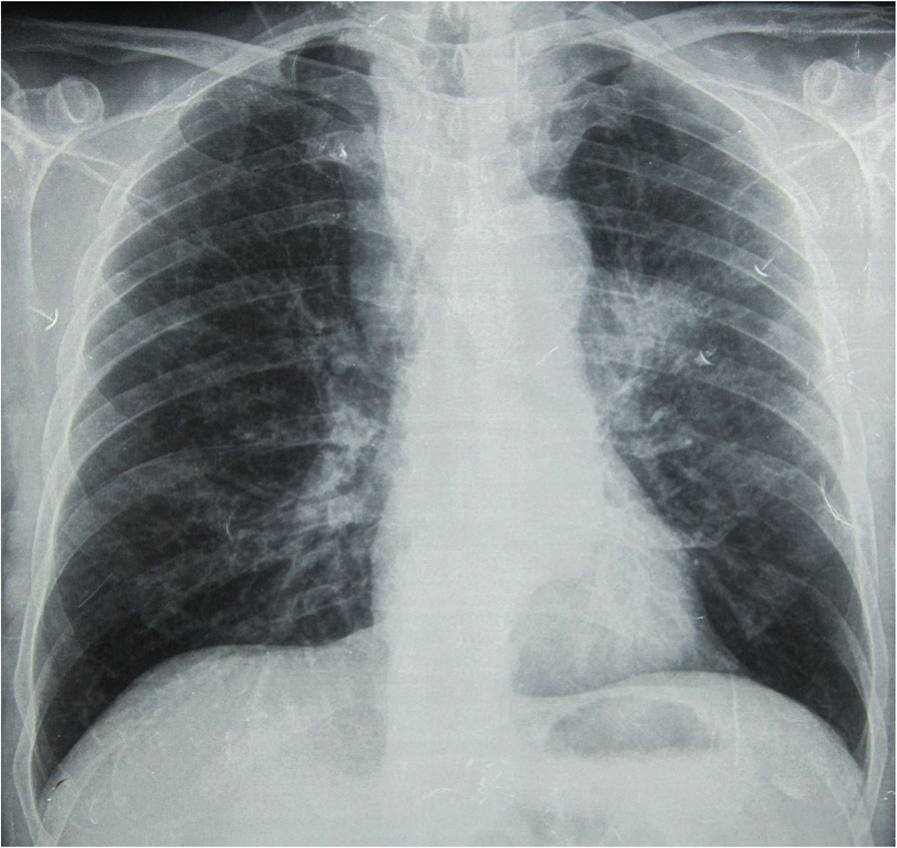
Chest x-ray on presentation, showing mediastinal widening and left parahilar infiltrate

Frontal chest x-ray revealed a mediastinal widening probably due to grossly enlarged right paratracheal and left paratracheal nodes as well as a mild left parahilar infiltrate with irregular borders (Fig. 1).

Electrocardiogram was in sinus rhythm, with a heart rate at 90 bpm. PR and QT intervals were normal, axis at 90°, without ST segment abnormalities or other waves and intervals. Blood tests showed an elevated white cell count exceeding 21,000 cells/mm^3^ with a lymphocyte count amounting to 13,750 and mild thrombocytosis. An elevated C reactive protein level (41 mg/l) was also noted, hinting to a biological inflammatory syndrome. Tests for hemostasis and blood biochemistry yielded a normal result. Lipid tests revealed hypertriglyceridemia (2.48 g/l). Urinalysis showed proteinuria (400 mg per day) with albumin presence, as well as hematuria. Serum protein electrophoresis was normal though.

**Fig. 2:**
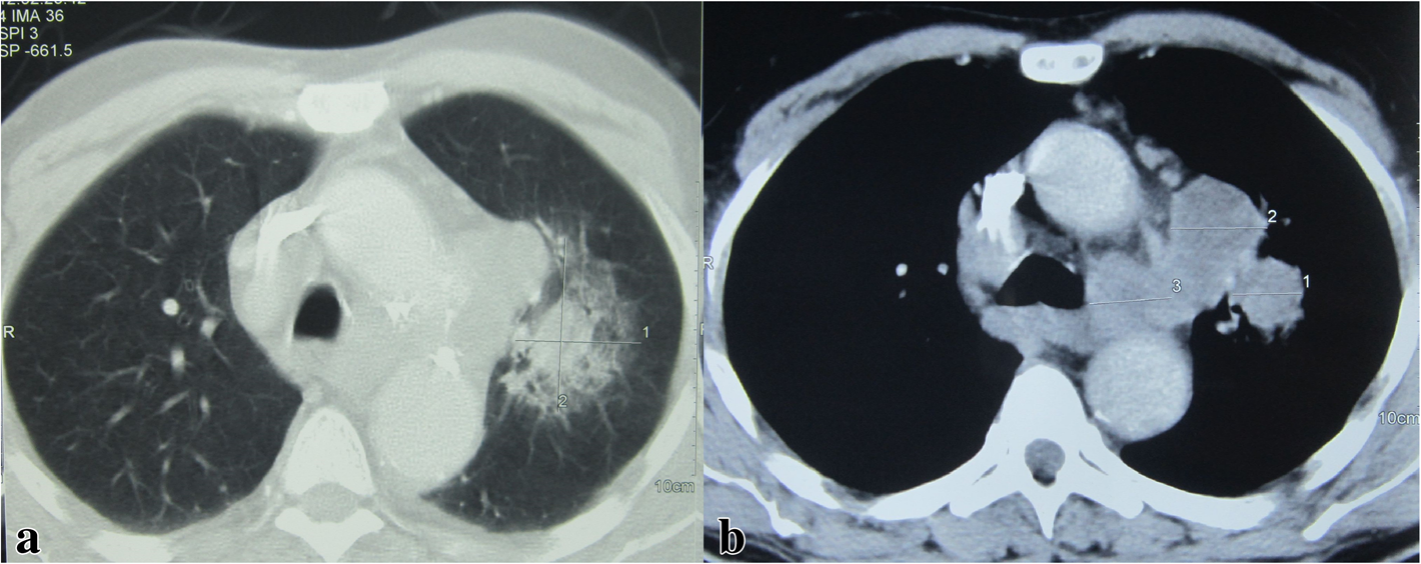
Initial chest CT. Attenuation in the left upper lobe and contralateral nodule on the parenchymal window (**a**). Bilateral adenopathy on the mediastinal window (**b**)

Chest x-ray findings urged us on performing a chest computed tomography (CT) scan. On the mediastinal window, it showed bilateral mediastinal and axillary lymphadenopathies, as well as enlarged supraclavicular lymph nodes on the left side. On the parenchymal window, CT revealed an ill-defined heterogeneous attenuation in the left upper lobe with blurred margins, as well as a solitary contralateral pulmonary nodule (Fig. 2). Abdominal and pelvic CT showed an adenoma of the left adrenal gland, bilateral simple kidney cysts and a subrenal aortic aneurysm. Brain CT scan showed no anomalies. Bone scintigraphy showed no evidence of osseous metastatic disease.

Hepatitis B and C serologic tests were negative. Immunological blood tests (antinuclear antibodies, extractable nuclear antigen antibodies, anti-dsDNA, anti-neutrophil cytoplasmic antibodies, cryoglobulin) were negative. Total immunoglobulins E level was normal.

Looking for histological evidence of a potential tumoral process, fibrobronchoscopy was performed, showing a mild bronchial stenosis of the upper lobe branch and thickening of the bifurcation between culmen and lingula branches with no evidence of endoluminal tumor. Aspiration cytology was benign. Bronchial biopsy examination could not prove malignancy, showing an inflammatory mucosa.

We eventually came to terms with performing a surgical biopsy of Virchow’s node. Pathology slides showed a B cell lymphomatous process that might tally with lymphocytic lymphoma of CLL type (tumoral subtype of old Kiel classification). Immunohistochemistry confirmed the B phenotype with CD5, CD20 and CD23 expression and a Ki-67 index exceeding 50% which heralds a transformation into diffuse large B cell lymphoma (DLBCL), also known as Richter syndrome (RS) or transformation. Additionally, one of the nodes was infiltrated by a malignant epithelial process with poorly differentiated large tumoral cells, hinting to a non-small cell carcinoma (NSSC). The latter were cytokeratin, TTF1, Napsin A positive and p40 negative in immunohistochemistry, which corroborated nodal metastatic disease of an adenocarcinoma. Taking into consideration that no clinical or radiological signs apart from the left upper lobe pulmonary lesion were found, we concluded to a pulmonary origin of the carcinomatous process thus making the diagnosis of primary adenocarcinoma of the lung.

Flow cytometry immunophenotyping was carried out showing normal proportions of B cell lymphocytes (CD19+ CD5- CD20+ CD22+) and T cell lymphocytes (CD3+ CD5+), amounting to 6% and 80% respectively, which are normal proportions for B and T populations.

Bone marrow aspiration and biopsy were then performed. Pathology examination showed massive infiltration of medullary spaces by small-sized lymphocytes with a few large atypical prolymphocytic cells, highly suggestive of CLL, which corroborates the lymph node biopsy examination findings. Besides, BCR-ABL transcript molecular testing was negative.

The diagnosis of metastatic adenocarcinoma of the lung associated with RS was made. Treatment consisted in chemotherapy, associating carboplatin, docetaxel and cyclophosphamide in the scope of a multidisciplinary approach.

**Fig. 3:**
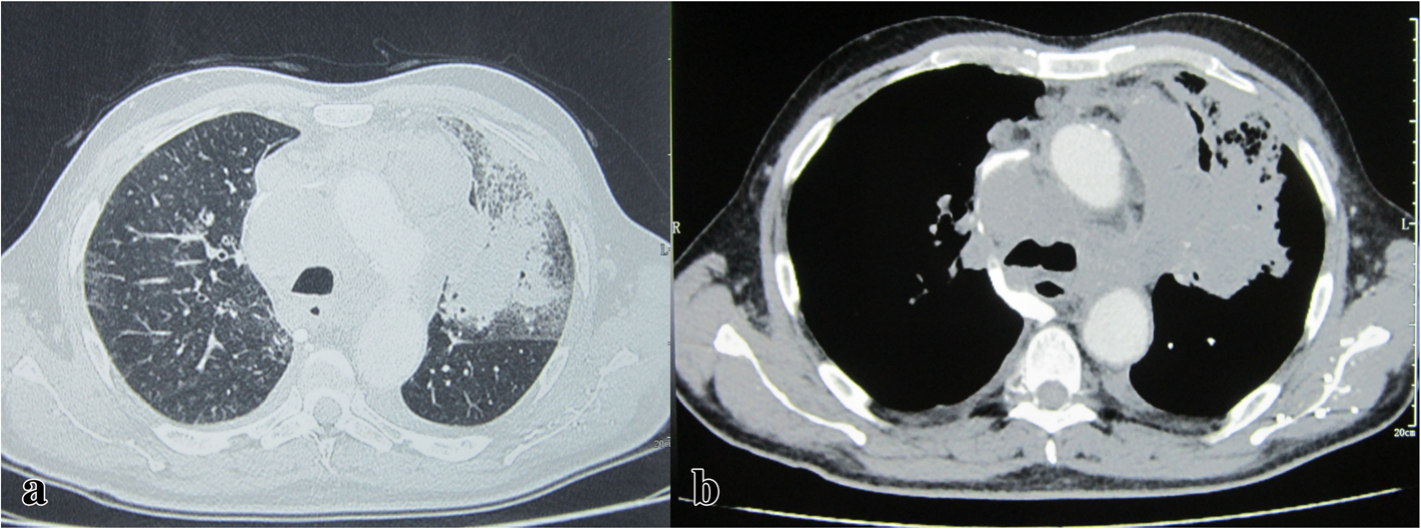
Control chest CT. Extension of the left upper lobe lesion on the parenchymal window (**a**). Increase in lymph nodes enlargement and superior vena cava obstruction on the mediastinal window (**b**)

**Fig. 4:**
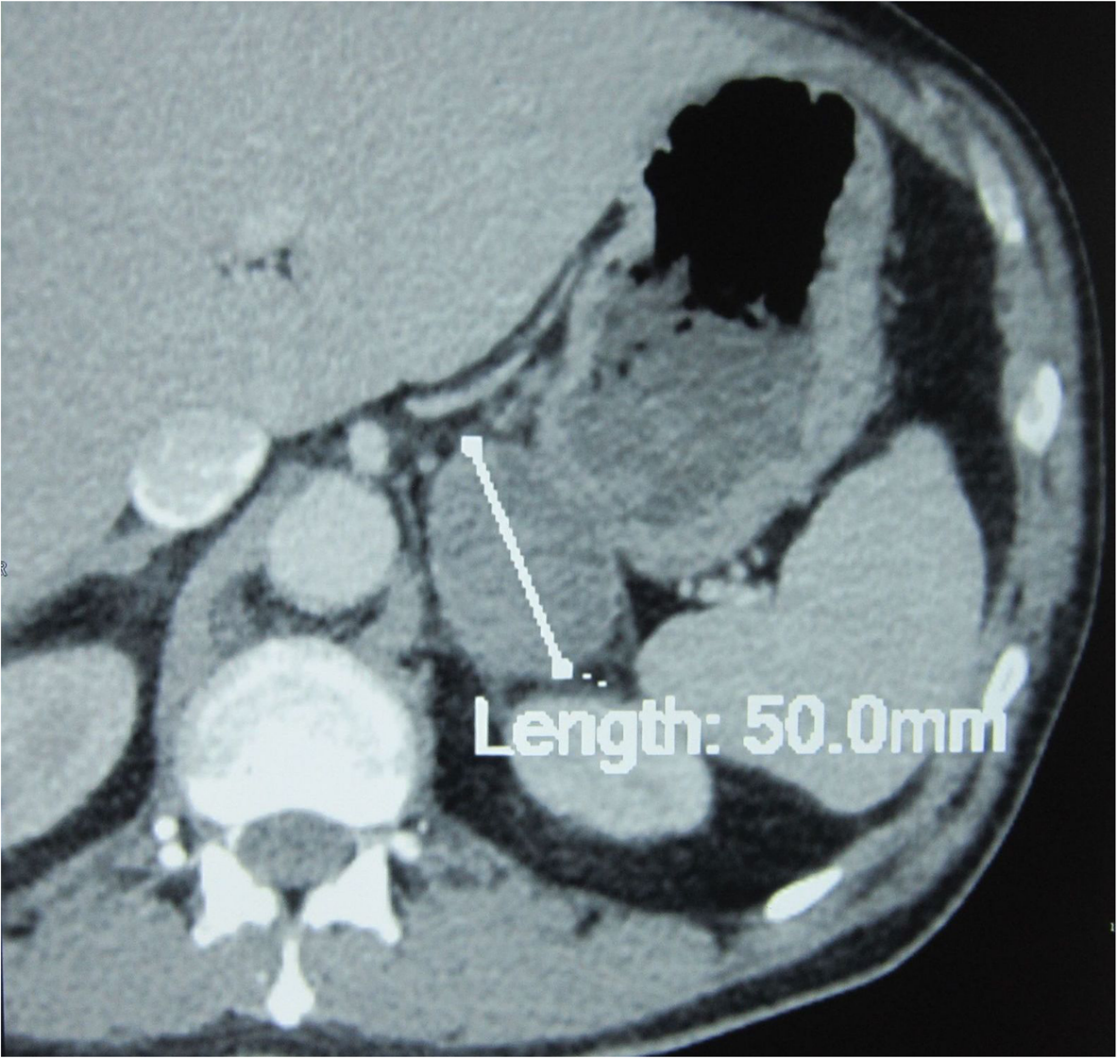
Control abdominal CT showing left adrenal gland metastasis

The course was unfavorable since the first days of therapy. In fact, the patient’s condition worsened swiftly after the first course of chemotherapy as he presented with severe dyspnea in relation to a superior vena cava (SVC) syndrome. CT showed an obstruction of the SVC, a major increase in the mediastinal lymph nodes enlargement, an extension of the left upper lobe mass as well as a left adrenal gland metastasis (Figs. 3 and 4). Echocardiography revealed a small pericardial effusion.

These findings urged us on prescribing corticosteroids and palliative radiation therapy. Within a fortnight, the patient showed further signs of deterioration with the onset of atrial fibrillation and a severe fatal infectious process.

## Discussion

CLL is a disorder characterized by a progressive accumulation of functionally incompetent monoclonal lymphocytes. It is the most common form of chronic lymphoproliferative disease [1]. Unlike our case, it usually occurs in the elderly, with a median age of 72 years at presentation [2].

RS is a unique and challenging complication of CLL. It bears an unfavorable prognosis since it is characterized by an aggressive presentation, chemotherapy-resistance and very poor survival [1]. The World Health Organization (WHO) defined it as an aggressive lymphoma arising on the background of known CLL [3]. In fact, most patients with RS transform from CLL to activated B cell (ABC) type DLBCL, with a minority turning into classical Hodgkin's lymphoma or other non-Hodgkin's lymphomas [4]. The features of this syndrome were first described by Maurice Richter in 1928 [5]. The term RS was coined in 1964 when Lortholary et al. reported a series of patients with CLL who developed DLBCL [6]. Recently published data has shown that RS arose in 2–10% of CLL patients during the course of their disease at a rate of approximately 0.5% to 1% *per annum* [7]. Besides, RS displays high genomic complexity and the acquisition of new genetic abnormalities is yielding more aggressive forms of the disease. In this respect, recent studies have shed some light on key genetic actors and it is now established that approximately 20% of RS cases emanate from a distinct clone of the underlying CLL [8].

Patients with usual signs of CLL (weight loss, fever, night sweats, muscle wasting and increasing hepatosplenomegaly and lymphadenopathy) are suspected of transformation when disproportionate weight loss, rapidly growing and/or asymmetrical lymphadenopathy or extranodal masses occur. An elevated lactate dehydrogenase level > 1.5 times the upper limit of normal or an elevated serum beta-2 microglobulin level > 2 mg/L are also alarming signs. Hypercalcemia, new onset of absolute lymphocytosis ≥5.10^9^/L, and thrombocytopenia <100.10^9^/L often accompany RS but do not seem to be reliable predictors of the disease [9]. In our patient, the presence of unilateral supraclavicular lymphadenopathy (Virchow’s node) was suggestive of the transformation into RS and histologic examination of this site yielded the diagnosis. However, no other relevant clinical or biological abnormalities were noted, which highlights how sneaky this syndrome can be.

The development of second malignancies in CLL cases, a *fortiori* with a RS transformation, is a hardly ever seen occurrence, and this gives further relevance to the present case. Prior studies have reported significantly increased risks of site-specific second cancers occurrence among patients with CLL [10–12]. Kaposi sarcoma, malignant melanoma and cancers of the larynx and the lung are the most frequently observed [12–14]. According to a recent study, patients aged less than 55 seem to have a greater risk of secondary solid tumors compared to the older patients [15]. This is in contrast with our case as the patient reported here is aged 56. An earlier study yielded similar results by showing that the relative risk of lung cancer did not vary by gender, or time of follow up, but was higher in younger (<60 years) than in older (70–79 years) age-groups [16].

Approximately 2% of patients with CLL develop lung carcinoma. According to Parekh et al., lung carcinoma is diagnosed a decade after CLL. Patients who develop both diseases die of lung carcinoma rather than of CLL. In our patient, the diagnosis of lung cancer was concomitant with that of CLL/RS which is uncommon [13]. The mysterious relationship between the two diseases is difficult to crack given the excess of smokers among CLL patients. Both non-small cell and small cell lung cancers are found, though adenocarcinoma is more common [17], a subtype highlighted in the present case.

Early work showed that lung cancer development is associated with immunological impairment in CLL patients [18, 19]. These defects seem to involve all cellular components of the cellular immune system, including quantitative and qualitative aspects of the normal B-cell pool, T cell subsets, natural killer cells and dendritic cells. Interestingly, B CLL cell has an impaired antigen-presenting capacity and can render the T cell anergic [18, 20]. On a biogenetic level, HER-2/neu overexpression - which is independent of smoking - has lately received significant attention as a culprit in the development and progression of lung cancer in patients with CLL [21].

Given its high resistance to conventional chemotherapy, treating CLL/RS even more so when it is complicated by second malignancies such as lung cancer, is a hard task. Further research aiming to target chemo-resistant DLBCL and the molecular abnormalities driving this transformation is eagerly awaited.

## Conclusions

In light of this case of rare occurrence, it might be sensible to consider heightened surveillance of CLL patients for second solid tumors, particularly lung cancer, and Richter’s transformation since the latter complications are a turning point in the course of the disease. Early diagnosis might yield a further understanding of its hallmarks and help grasp the potential impairment of immune system in the pathways of the disease. Doing so would smooth the way for a better care.

## Abbreviations

CLL: Chronic lymphocytic leukemia
CT: Computed tomography
DLBCL: Diffuse large B cell lymphoma
NOS: Not otherwise specified
NSCC: Non-small cell carcinoma
RS: Richter syndrome
SVC: Superior vena cava
